# Antibacterial activity of *Cinnamomum cassia* L. essential oil in a carbapenem- and polymyxin-resistant *Klebsiella aerogenes* strain

**DOI:** 10.1590/0037-8682-0032-2020

**Published:** 2020-10-05

**Authors:** Nathalie Gaebler Vasconcelos, Késia Esther Silva, Júlio Croda, Simone Simionatto

**Affiliations:** 1 Universidade Federal da Grande Dourados, Laboratório de Pesquisa em Ciências da Saúde, Dourados, MS, Brasil.; 2 Universidade Federal da Grande Dourados, Hospital Universitário de Dourados, Dourados, MS, Brasil.; 3 Fundação Oswaldo Cruz, Campo Grande, MS, Brasil.; 4 Universidade Federal do Mato Grosso do Sul, Campo Grande, MS, Brasil.

**Keywords:** Drug resistance, Cinnamon oil, Carbapenem-resistant Enterobacterales, Gram-negative bacteria, Klebsiella aerogenes, Polymyxin resistance

## Abstract

**INTRODUCTION:**

Essential oils can serve as novel sources of antibiotics for multidrug-resistant bacteria.

**METHODS:**

The multidrug-resistance profile of a *Klebsiella aerogenes* strain was assessed by PCR and sequencing. The antibacterial activity of *Cinnamomum cassia* essential oil (CCeo) against *K. aerogenes* was assessed by broth microdilution and time-kill methods.

**RESULTS:**

*K. aerogenes* showed high antibiotic resistance. The genes *bla*_KPC-2_, *ampC*, *bla*_CTX-M-15_, *bla*_OXA-1_, and *bla*_TEM_ were present. CCeo exhibited an inhibitory effect with a minimum inhibitory concentration of 17.57 μg/mL.

**CONCLUSIONS:**

The antibacterial activity of CCeo makes it a potential candidate for treating carbapenem- and polymyxin-resistant *K. aerogenes* strains.

The emergence of multidrug-resistant bacteria is an important public health issue. *Klebsiella aerogenes*, previously known as *Enterobacter aerogenes*, has become an important opportunistic pathogen that often causes hospital-acquired infections such as pneumonia, urinary tract infections, bacteremia, and surgical site infections^1^. Worldwide, the number of infections caused by *K. aerogenes* strains with acquired resistance to most classes of commercially available antimicrobial agents, including carbapenems and polymyxins, is rising^2^. Carbapenems are often used to treat serious infections caused by *K. aerogenes*, especially those that produce AmpC cephalosporinases or extended-spectrum β-lactamases (ESBL)^1^, but carbapenem resistance can also arise. This scenario has led us to reconsider polymyxins as valuable therapeutic options. However, in addition to carbapenem resistance, various genera have demonstrated the ability to acquire polymyxin resistance^3^.

Carbapenemase-producing pathogens that are also resistant to polymyxins are a matter of concern, as these characteristics drastically reduce therapeutic options^4^. Carbapenem- and polymyxin-resistant Enterobacterales are considered critical by the World Health Organization, which has thus listed research into and development of new antibiotics as a high priority.

Polymyxin-resistant *K. aerogenes* strains have been identified and are being increasingly reported^5^. This highlights the urgency to find novel treatment options for carbapenem- and polymyxin-resistant bacterial infections. Molecular analysis of such strains may help improve our understanding of the antimicrobial resistance mechanisms and allow for the development of new and effective antibiotics.

With an increasing number of antibiotics rendered ineffective, the study of medicinal plants as potential sources of novel antibiotics, especially for the treatment of multidrug-resistant infections, has increased^5^. Plant-derived essential oils contain potent natural agents, known as secondary metabolites, that have been used for hundreds of years against pathogens^6^. An important characteristic in essential oils is their hydrophobicity, which allows them to interact with the lipids in the bacterial cell membrane and disturb the bacterial structure, thereby making it more permeable and leading to the leakage of ions and various molecules from the bacterial cells. Although the extravasation of cellular components can be tolerated without loss of viability, further loss of cellular content or the loss of critical molecules and ions can lead to cell death^4^. *Cinnamomum cassia* L. (Lauraceae) essential oil (CCeo), popularly known as cinnamon, contains a wide variety of secondary metabolites and has been shown to exhibit antimicrobial effects^7^. In this study, the antimicrobial activity of CCeo in the inhibition of a carbapenem- and polymyxin-resistant *K. aerogenes* strain was evaluated.

CCeo is a yellow to yellow brown clear oily liquid, extracted from leaves, bark, and branches by steam distillation, with a density of 1.053 g/cm^3^. CCeo originated in China, and this compound was acquired commercially from Ferquima (Vargem Grande Paulista, SP, Brazil) (CAS number: 84961-46-6). According to the chromatographic technical report provided by the supplier, cinnamaldehyde was the most prevalent compound (87.6%), followed by α-humulene (3.1%), γ-elemene (2.5%), borneol (1.5%), cinnamic acid (0.7%), benzaldehyde (0.5%), and eugenol (0.4%), and other minor components (3.7%) (Figure 1).

**FIGURE 1:**
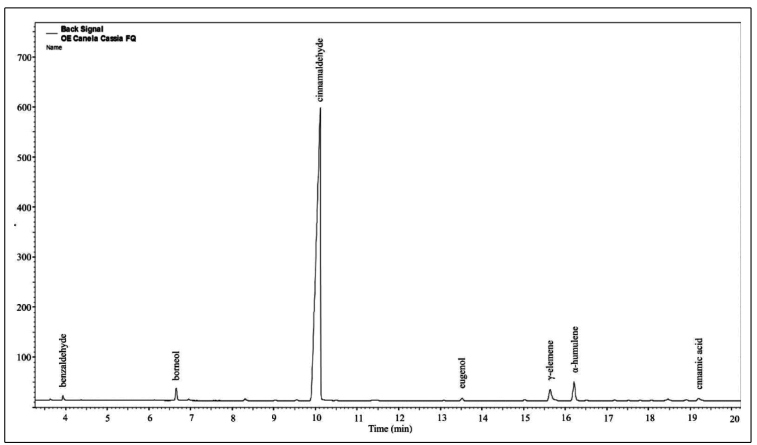
The chromatographic profile obtained with high-resolution gas chromatography for *Cinnamomum cassia* oil using an AGILENT 7820A Gas Chromatograph. Column: HP-5 30 m × 0.32 mm × 0.25 μm (AGILENT). Temp.: Column: 70°C (0 min), 3°C/min at 240°C. Injector: 240°C Split: 1/50. FID detector: 250°C. Vol. injection time: 1 µL (conc 1.0% in chloroform). Vany Ferraz, Chemistry Department, Universidade Federal de Minas Gerais. Requested by Ferquima (Vargem Grande Paulista, SP, Brazil).

A clinical isolate of a carbapenem- and polymyxin-resistant *K. aerogenes* was identified in September 2015 in a microbiological vigilance culture (nasal swab) at a tertiary Brazilian hospital. The isolate was identified using BD Phoenix 100 (Becton Dickinson, Franklin Lakes, NJ) and the antimicrobial susceptibility was determined by broth microdilution and by using BD Phoenix 100. Preliminary screening for the presence of carbapenemase was performed using the modified Hodge test^8^. The study was conducted with the approval of the Research Ethics Committee of the Universidade Federal da Grande Dourados (Process no. 877.292/2014).

Polymerase chain reaction (PCR) and sequencing were performed to determine the presence of the carbapenems-resistance genes *bla*_CTX-M-1-like_, *bla*_CTX-M-2-like_, *bla*_CTX-M-8-like_, *bla*_CTX-M-14-like_, *bla*_GES-like_, *bla*_GIM-like_, *bla*_IMP-10_, *bla*_IMP-like_, *bla*_KPC-2_ *, bla*_NDM-like_, *bla*_OXA-23_, *bla*_OXA-48-like_, *bla*_SHV-like_, *bla*_SIM-like_, *bla*_SME-like_, *bla*_SPM-like_, *bla*_TEM-like_, and *bla*_VIM-like_ ^2^, and the polymyxin B-resistant gene *mcr-1* ^5^.

Genomic DNA was extracted from a fresh culture. The concentration and purity of the DNA were determined with a Qubit 2.0 fluorometer using the dsDNA BR Assay Kit (Life Technologies, Carlsbad, CA). A sequencing library was prepared using the Nextera library kit (Illumina, San Diego, CA). The DNA sample was sequenced using the Illumina MiSeq Platform (Illumina), as described by Dung et al.^9^ Readings were mapped to a reference sequence, and species identification was confirmed using the taxonomic classification system of Kraken^10^. The reading set was assembled using SPAdes version 3.6.1 and annotated using the software tool Prokka^11^. The reading set was also screened for known alleles using a reading mapping approach with SRST2, the Short Read Sequence Typing for Bacterial Pathogens. For the acquired resistance genes, the Antibiotic Resistance Gene-ANNOTation (ARG-ANNOT) database was used^12^.

The minimum inhibitory concentration (MIC) of CCeo was determined by broth microdilution and by using a resazurin colorimetric assay^13^. The tested concentration range of CCeo was 72,000 μg/mL-2.197 μg/mL. The time-kill method was performed using the broth macrodilution technique^14^. The concentration of CCeo and polymyxin B tested represented the MIC value. Time-kill studies were performed with approximately 1.5 × 10^6^ CFU/mL. A positive control of tigecycline was used at twice the MIC value, since the studied strain had an intermediate sensitivity to this antibiotic and there was no sensitivity to other pharmacological classes. A negative control of brain heart infusion broth was used for the bacterial strain, and saline was used as sterility control.

The *K. aerogenes* strain has an ESBL-, carbapenem- and polymyxin-resistant profile (Table 1). The genomic data assembled using ResFinder identified 17 resistance genes. The metallo-β-lactamase resistance genes identified were *ampC*, *bla*_CTX-M-15_, *bla*_KPC-2_, *bla*_OXA-1_, and *bla*_TEM_. Other resistance genes observed in this strain, including *aac3, aac6, aphA6, catB4, dfrA8, dfrA14, qnrB1, strA, strB, sul2, tetA*, and *tetR*, conferred resistance to aminoglycosides, chloramphenicol, trimethoprim, fluoroquinolones, streptomycin, sulfamethoxazole, and tetracycline. The genes *bla*_CTX-M-1-like_, *bla*_CTX-M-2-like_, *bla*_CTX-M-8-like_, *bla*_CTX-M-14-like_, *bla*_GES-like_, *bla*_GIM-like_, *bla*_IMP-10_, *bla*_IMP-like_, *bla*_NDM-like_, *bla*_OXA-23_, *bla*_OXA-24,_ *bla*_OXA-48,_ *bla*_OXA-48-like_, *bla*_OXA-51,_ *bla*_OXA-58,_ *bla*_SHV-like_, *bla*_SIM-like_, *bla*_SME-like_, *bla*_SPM-like_, *bla*_TEM-like_, *bla*_VIM-like_, and *mcr-1* were not detected.

**TABLE 1:** Antimicrobial susceptibility patterns (MICs in µg/mL).

Antibiotics	MIC (interpretation)
Amikacin	32 (I)
Aztreonam	> 16 (R)
Ceftazidime	> 16 (R)
Cefepime	> 16 (R)
Ceftriaxone	> 32 (R)
Ciprofloxacin	> 2 (R)
Ertapenem	> 4 (R)
Gentamicin	≤ 2 (R)
Imipenem	8 (R)
Meropenem	8 (R)
Piperacillin/Tazobactam	> 64 (R)
Polymyxin B	> 4 (R)
Tigecycline	2 (I)
Sulfamethoxazole/Trimethoprim	> 4 (R)

**(I):** intermediate; **(R):** resistance; **MIC:** minimum inhibitory concentration.

Antimicrobial resistance to carbapenems and polymyxin B was confirmed. Molecular analysis was used to identify the *bla*_KPC-2_ gene, elucidating the mechanism of carbapenem resistance. Sequencing also allowed us to identify other metallo-β-lactamase genes, such as *ampC*, *bla*_CTX-M-15_, *bla*_OXA-1_, and *bla*_TEM_, which provide *K. aerogenes* with a wide range of resistance mechanisms, possibly capable of transferring related genes, leading to an increase resistance to antibiotics, thus making it very difficult to treat infections caused by this bacterial strain. Resistance to polymyxin B due to the presence of *mcr-1* gene was not considered. However, sequencing results showed alterations in the *phoP* gene, indicating a potential mechanism of mutational colistin resistance in this strain.

CCeo exhibited an inhibitory effect against the carbapenem- and polymyxin-resistant *K. aerogenes* strain even at a low concentration (0.0019% v/v) and had a MIC value of 17.57 μg/mL. The survival curve of the strain showed a decrease in viable cell counts over time (Figure 2). CCeo treatment decreased cell counts by approximately 5 log_10_ CFU/mL. The results showed total inhibition of the carbapenem- and polymyxin-resistant *K. aerogenes*, with cell counts reduced to zero after 24 h of CCeo treatment. Furthermore, polymyxin B showed no activity for up to 24 h. Tigecycline was used at twice the MIC value as a positive control and was found to inhibit the strain successfully within 24 h.

**FIGURE 2:**
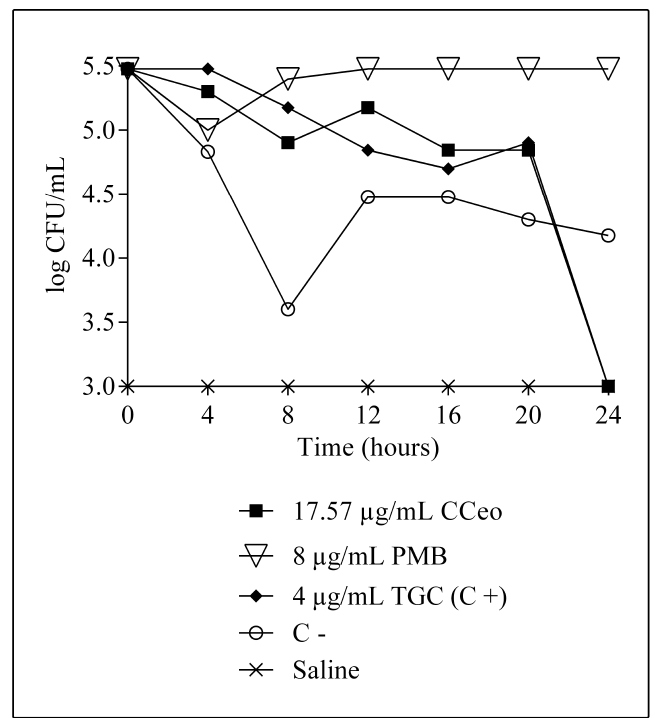
Time-kill curves with CCeo against a carbapenem and polymyxin-resistant *K. aerogenes* strain. **CCeo:** *Cinnamomum cassia* L. essential oil; **MIC:** minimum inhibitory concentration; **PMB:** polymyxin B; **TGC:** tigecycline; **C+:** positive control (tigecycline at twice the MIC value); **C-:** negative control (*K. aerogenes* and brain heart infusion broth).

CCeo demonstrated promising antibacterial properties against the carbapenem- and polymyxin-resistant *K. aerogenes* strain, a bacterial strain resistant to almost all classes of clinical antibiotics, with a low MIC and a consistent action. To the best of our knowledge, this is the first report on the antibacterial activity of CCeo against multidrug-resistant *K. aerogenes*. In addition, to determine MIC values, an improved time-kill method was used, which allowed us to verify the speed of the inhibition activity of CCeo on the studied bacteria, to enhance our understanding of the underlying mechanism of action of CCeo.

Our results suggest that the CCeo antibacterial action mechanism is complex. Several pathways may be involved in the inhibitory effects of this oil against bacterial cells, making it difficult to elucidate them all. Multiple experiments are necessary in order to evaluate all possible mechanisms and provide an insight into the molecular actions of CCeo. The antimicrobial activity of essential oils may involve single or multiple targets. Therefore, the mechanisms of action cannot be attributed to a unique site, but rather to a cascade of reactions involving the entire bacterial cell^6^.

Regarding CCeo toxicity, the major components of *C. cassia* are considered to be non-toxic and safe, with no acute or chronic toxicity, no mutagenicity or genotoxicity, and no carcinogenicity detected in mammalian studies^15^, making CCeo even more suitable for use as raw material in drug development.

Effective antimicrobial activity was observed at low concentrations, indicating that CCeo is a favorable candidate for *in vivo* studies in animal models to demonstrate its clinical applicability for the future development of antimicrobial treatments. Our findings fall in line with the critical need for novel sources of antibiotics to address an increasing incidence of drug-resistant pathogens, especially multidrug-resistant bacteria. However, this study had some limitations. Studies on other multidrug-resistant bacteria and *in vivo* studies are necessary to better understand the mechanisms of action of CCeo.

Our data reveal that the CCeo had an antibacterial effect, making it an interesting candidate for the development of therapeutic options against carbapenem- and polymyxin-resistant *K. aerogenes* strains. Further studies on CCeo activity in animals infected with multidrug-resistant bacteria are essential in order to further improve our understanding of its actions and establish its efficacy.
